# Prevalence and Risk Factors of Oropharyngeal Dysphagia in Newly Diagnosed Head-and-Neck Cancer Patients

**DOI:** 10.3390/cancers17010009

**Published:** 2024-12-24

**Authors:** Michelle G. M. H. Florie, Monse W. M. Wieland, Walmari Pilz, Rosanne Partoens, Bjorn Winkens, Ann Hoeben, Nathalie Rommel, Laura W. J. Baijens

**Affiliations:** 1 Department of Otorhinolaryngology, Head and Neck Surgery, Maastricht University Medical Center, P.O. Box 5800, 6202 AZ Maastricht, The Netherlands; 2 GROW—Research Institute for Oncology and Reproduction, Maastricht University, P.O. Box 6200, 6211 LK Maastricht, The Netherlands; 3 Department of Neurosciences, ExpORL, Deglutology, University of Leuven, 3000 Leuven, Belgium; 4 Department of Methodology and Statistics, Maastricht University, P.O. Box 6200, 6211 LK Maastricht, The Netherlands; 5 Care and Public Health Research Institute—CAPHRI, Maastricht University, P.O. Box 6200, 6211 LK Maastricht, The Netherlands; 6 Division of Medical Oncology, Department of Internal Medicine, Maastricht University Medical Center, P.O. Box 5800, 6202 AZ Maastricht, The Netherlands; 7 Department of Gastroenterology, Neurogastroenterology and Motility, University Hospitals Leuven, 3000 Leuven, Belgium

**Keywords:** oropharyngeal dysphagia, head-and-neck cancer, malnutrition, screening, deglutition disorders

## Abstract

Background: Head-and-neck cancer (HNC) can cause oropharyngeal dysphagia (OD). Early identification of OD in newly diagnosed HNC patients is important to better prepare patients for their cancer treatment trajectory. The aim of this study is (1) to assess the prevalence of OD in HNC patients within three weeks before the start of cancer treatment and (2) to investigate which demographic and oncological characteristics may be risk factors associated with the risk of OD at baseline. Methods: Patients (N = 225) completed the Eating Assessment Tool-10 (EAT-10) and Short Nutritional Assessment Questionnaire (SNAQ). Logistic regression analysis was conducted to examine the association between OD versus demographic and oncological characteristics. Results: A total of 21.3% (proportion 0.213; 95% CI 0.163–0.274) of the patients were at risk for OD. After correction for age, Charlson Comorbidity Index (CCI) grade, and primary tumor location, a significant association was found between advanced-stage cancer versus the risk of OD. Additionally, post hoc analysis revealed a significant association between the risk of malnutrition versus the risk of OD. Conclusions: Approximately one-fifth of all newly diagnosed HNC patients are at risk of OD, with advanced-stage cancer and malnutrition emerging as significant risk factors of OD. These findings empower health professionals toward more effective screening and management of a patient’s risk profile before the start of HNC treatment.

## Simple Summary

To our knowledge, no studies have been published on systematic screening for oropharyngeal dysphagia (OD) in all newly diagnosed head-and-neck cancer (HNC) patients irrespective of cancer stage and primary tumor location. This knowledge gap has led to uncertainty regarding the prevalence of OD within this population. Moreover, the prevalence of OD and its relationship with demographic and oncological characteristics can guide us to more effective screening and management of a patient’s risk profile before the start of cancer treatment. This study unveiled that approximately one-fifth of all newly diagnosed HNC patients are at risk of OD before the start of cancer treatment, with advanced-stage cancer and malnutrition emerging as significant risk factors. These findings equip health professionals to prioritize closer monitoring and tailored interventions to prepare patients with a risk of OD and OD-related complications for their entire cancer treatment trajectory.

## 1. Introduction

Head-and-neck cancer (HNC) refers to a group of malignancies that arise in the nasal cavity, paranasal sinuses, oral cavity, pharynx, larynx, and salivary glands [1]. Worldwide, HNC is one of the most frequently occurring malignancies, with high mortality rates, accounting for 4.6% of cancer deaths [2]. In the Netherlands, more than 3000 patients are diagnosed with HNC annually [3]. It is well known that HNC patients often experience swallowing problems, which may be caused by the cancer and/or cancer treatment [4,5]. Oropharyngeal dysphagia (OD) involves disturbances in the sensibility, mobility, and/or neuromuscular coordination of the upper aerodigestive tract during the complex oral preparatory, oral, pharyngeal, and esophageal phases of swallowing [6,7]. Additionally, HNC patients often have a pre-existing compromised nutritional status due to an unhealthy lifestyle characterized by excessive alcohol and tobacco consumption, and a diet lacking various nutrients [8]. It has been suggested that impaired nutritional status before the start of cancer treatment may affect swallowing function due to sarcopenia and a malnourished skeletal muscle status, even in the presence of a normal body mass index (BMI) [9,10]. The presence of baseline OD in newly diagnosed HNC patients may impact the choice of cancer treatment modalities and affect treatment outcomes.

Predicting which HNC patients will have a higher baseline risk of OD and OD-related complications, such as malnutrition, aspiration pneumonia, sepsis, and even mortality, is challenging [7]. For patients who have just been informed of their cancer diagnosis, it is not self-evident that they will spontaneously report swallowing problems, as they are usually in an existential crisis, focusing on survival. However, early identification and intervention for OD and malnutrition, starting at the initial diagnosis of HNC, are paramount. This enables the referral of patients to allied health professionals for a comprehensive assessment of OD and malnutrition, and the initiation of prehabilitation before the start of cancer treatment, and ensures that patients are better prepared to undergo cancer treatment. This proactive approach can reduce the risk of toxicity and complications from cancer treatment, prevent OD-related sequels, and ultimately improve oncological outcomes, including survival rates and overall health-related quality of life [11,12,13]. Currently, there is no consensus on which tools should be used for screening OD in newly diagnosed HNC patients. Considering that the period before the start of cancer treatment is typically filled with numerous diagnostic tests for cancer staging, it is essential to efficiently manage the identification of patients at risk for OD by selecting an acceptable, reliable, and easy-to-perform screening method.

Currently, very few studies report on the prevalence of being at risk of OD in newly diagnosed HNC patients [14,15]. Cates et al. (2022) used the patient-reported Eating Assessment Tool-10 (EAT-10) [16] and found that 83 of the 144 HNC patients (58%) had an abnormal EAT-10 score before initiating upfront or postoperative chemoradiation for advanced-stage cancer [14,15]. Similarly, about a quarter (26.2%) of the 128 HNC patients were at risk of OD (EAT-10 ≥ 3) before the start of cancer treatment in the study by Wieland et al. (2023) [14,15]. To our knowledge, no studies have been published on the systematic screening for OD in all newly diagnosed HNC patients, irrespective of cancer stage, primary tumor location, or cancer treatment modality, nor on the relationship between OD and its potential risk factors at baseline, i.e., before the start of cancer treatment. Factors such as aging, lifestyle issues, and comorbid conditions, which are common in the HNC population, are potential risk factors for the development of OD already before the start of cancer treatment. Furthermore, understanding the prevalence of being at risk of OD and its relationship with demographic and oncological characteristics can help guide more effective screening and management of patients’ risk profile before the start of cancer treatment. This knowledge gap has created uncertainty regarding the baseline prevalence and risk factors of OD in this population, despite the critical importance of such information for optimal patient preparation. Effective preparation is essential for facilitating shared decision-making in selecting a feasible, personalized cancer treatment modality and for ensuring the successful completion of cancer treatment. Given the limited published data on baseline screening and assessment of OD in this population, a decision was made to implement systematic screening for OD in all newly diagnosed HNC patients in the present cohort study. This approach was further informed by clinical experience, which suggests that health professionals often rely on selective screening guided by “gut feeling” or intuition, particularly when patients appear thin, have a wet voice, or exhibit other signs of dysphagia. With this in mind, the aim of this study is twofold: (1) to assess the prevalence of OD in HNC patients within three weeks before the start of cancer treatment, and (2) to investigate which demographic and oncological characteristics may be risk factors associated with the risk of OD at baseline. It is hypothesized that a significant subset of newly diagnosed HNC patients are at risk of OD and that this risk may be associated with risk factors such as older age, a poor Charlson Comorbidity Index (CCI) grade, primary tumor location, and advanced-stage cancer.

## 2. Materials and Methods

### 2.1. Study Design and Participants

The participants in this prospective cohort study were newly diagnosed HNC patients who visited the Comprehensive Cancer Center of Maastricht University Medical Center+ (MUMC+) in the Netherlands between December 2021 and May 2023. The inclusion and exclusion criteria are displayed in Table 1. The study was approved by the medical ethics committee of the MUMC+ (METC 2022-3133).

### 2.2. Demographic and Oncological Data Collection

Demographic characteristics (age, sex, body mass index (BMI), tobacco and alcohol consumption, marital status, and occupation), oncological characteristics (primary tumor site, Tumor Node Metastasis (TNM) classification 8th edition [17], and cancer stage grouping), comorbidity, and performance status (PS) were extracted from the mandatory national healthcare registry, the “Dutch Head and Neck Audit” (DHNA), by two independent researchers. The DHNA monitors the quality of HNC care in all Dutch cancer centers and contains strictly protocolled data [18]. Comorbidity was obtained using the CCI grade [19]. The CCI is a validated tool used to assess the severity of patients’ comorbid condition by considering both the number and severity of predefined comorbidities based on the International Classification of Diseases (ICD-10). It provides a weighted comorbidity score that can predict short- and long-term outcomes, including functional outcomes, duration of hospital stay, and mortality. A CCI grade ranging from 0 to 3 was derived from the total CCI scores, with “0” indicating “no comorbid condition” and “3” indicating “a severe comorbid condition” [19]. The patient’s ability to perform activities of daily living (ADL) was measured using the PS according to the World Health Organization (WHO) scale [20]. This ordinal scale provides a score ranging from 0 to 4, where “0” represents “a fully active condition” and “4” “a completely disabled status.”

### 2.3. Screening for Oropharyngeal Dysphagia and Malnutrition

All consecutive newly diagnosed HNC patients underwent standardized OD and malnutrition screening within 10 days after the patient’s first visit and within three weeks before the start of cancer treatment. According to the Dutch SONCOS (Stichting Oncologische Samenwerking) Standardization report, cancer treatment should start within 30 days of the patient’s first visit [21]. The EAT-10 is a patient-reported questionnaire consisting of 10 items addressing OD-specific symptoms. Responses are given on a 5-point Likert scale, ranging from “0”, indicating “no swallowing problem”, to “4”, indicating “severe swallowing problems” [16,22]. The maximum total score is 40 points. A score of ≥3 indicates a high risk of self-perceived symptoms of OD. The Short Nutritional Assessment Questionnaire (SNAQ) and BMI were used to determine the risk of malnutrition based on the Dutch clinical practice guideline for malnutrition [23]. The validated Dutch SNAQ questionnaire consists of four items that assess unintentional weight loss, decreased appetite, and the use of complementary drinks or tube feeding [24]. The maximum total score is 7 points. A score of ≥2 indicates a high risk of malnutrition. BMI was calculated for all patients based on weight and height measured with the same equipment. BMI was scored as abnormal when BMI < 20 kg/m^2^ if age < 70 y or <22 kg/m^2^ if age ≥ 70 y. The justification of the selection of the EAT-10 as a screening tool for OD and its implementation in clinical practice as part of a multi-domain screening framework was described in a previous study [15]. The screening was performed by three oncology nurses with more than 10 years of clinical experience in HNC. Prior to the introduction of the screening protocol in everyday practice, the nurses followed intensive multiple-session training on the purpose of the screening and how to carry out the measurements in a standardized way.

### 2.4. Statistical Analysis

To determine the prevalence of being at risk of OD, descriptive statistics were conducted. Means with standard deviations (SDs) and medians with interquartile ranges (IQRs, 25th–75th percentile) were used to report normally and non-normally distributed numerical variables, respectively. Normality was assessed using histograms and Q-Q plots. Numbers and percentages were utilized to report the frequency distribution of categorical variables. Independent-sample *t*-tests, Mann–Whitney U-tests, Fisher’s exact tests, Pearson chi-square tests, and median tests were employed for group comparisons between patients included in the study and those excluded for not completing the EAT-10. To examine the relationship between the risk of OD versus demographic and oncological characteristics, the following steps were taken.

Firstly, descriptive data were reported using the full range of scores for each variable, i.e., not categorizing numerical variables. Subsequently, group comparisons of EAT-10 scores between dichotomized demographic and oncological characteristics (age, CCI grade, PS score, malnutrition risk, and cancer stage grouping) were performed using median tests and Pearson chi-square tests, as appropriate. The sample size of the present study was insufficient to encompass all the individual categories of the demographic, oncological, and screening variables in the multivariable logistic regression models. Therefore, to increase the number of observations per category, all categorical variables, except for primary tumor location, were dichotomized based on their validated cut-off values or based on clinical reasoning. For consistency reasons, the variable age was also dichotomized: those under 70 years old and those aged 70 and above. In the Netherlands, patients over 70 are typically excluded from platinum-based concurrent chemoradiation due to the associated toxicity, as many elderly patients have renal, cardiac, or other comorbidities. For the CCI grade, patients were divided into two groups: one with no or mild comorbid conditions (grade 0–1) and another with more severe comorbid conditions (grade 2–3). A PS cut-off of ≥2 was used to dichotomize this variable, consistent with previous studies that found scores of ≥2 being associated with poorer cancer treatment outcomes [25,26]. Based on the SNAQ and BMI scores, patients were divided into a group at risk of malnutrition (SNAQ ≥ 2 or BMI < 20 kg/m^2^ if <70 years old or BMI < 22 kg/m^2^ if ≥70 years old) and another group not at risk (SNAQ < 2 and BMI > 20 kg/m^2^ if <70 years old or >22 kg/m^2^ if ≥70 years old). For cancer stage grouping, patients were divided into early-stage (0–1–2) or advanced-stage cancer groups (3–4). Risk of OD was defined as an EAT-10 score ≥ 3.

Secondly, logistic regression analysis was conducted to examine the relationship between OD versus demographic and oncological characteristics. To avoid overfitting or unnecessary complexity in the model, we included only the variables that we hypothesized to have the strongest associations: age, CCI grade, cancer stage grouping, and primary tumor location. However, since group comparisons revealed an association between the risk of malnutrition and the risk of OD, an additional post hoc logistic regression analysis with correction for malnutrition risk was conducted to see whether the significant associations from the primary logistic regression analysis remained significant. It is important to note that dichotomizing variables can lead to a loss of information, reduced measurement precision, and decreased statistical power. To verify the consistency of the results of the logistic regression analysis when treating numerical variables as numerical (more power) and using the full range of the variable’s categories instead of dichotomizing them, sensitivity analysis was performed. If the findings from the sensitivity analysis align with those of the primary analysis and produce similar results, this confirms that dichotomization had minimal or no impact on the primary outcomes. Therefore, a sensitivity analysis using linear regression was conducted on the EAT-10 scores, considering their full range, to assess any potential loss of information resulting from dichotomization. A two-sided significance level of α = 0.05 was used for all statistical tests. All statistical analyses were performed in IBM SPSS statistics v.29 (IBM Corporation, Armonk, NY, USA).

## 3. Results

### 3.1. Study Design and Participants

Of the 266 newly diagnosed HNC patients who visited the Comprehensive Cancer Center, 225 completed the EAT-10 (84.6%). Several reasons contributed to incomplete EAT-10 questionnaires, including patients missing their appointment or not returning the questionnaire, refusal to participate in the screening, the unavailability of nursing staff, continuing the cancer treatment at another hospital, or the patient’s death.

### 3.2. Patient Demographic and Oncological Characteristics

Of the patients included in the study, 144 (64.0%) were male, and the mean age was 66.8 years (SD: 10.7). The oral cavity was the most common primary tumor site (N = 77; 34.2%). Most patients had a CCI grade of ≥1 (51.8%) and a PS score of ≥1 (32.1%), indicating a high prevalence of comorbidities and functional impairments. The mean BMI was 25.8 kg/m^2^ (SD: 5.0), and the median SNAQ score was 0.0 (IQR: 0–1), suggesting a low risk of malnutrition. Demographic and oncological characteristics are presented in Table 2. Additionally, univariable analysis was conducted to compare the demographic and oncological characteristics between two groups: patients who completed the EAT-10 and those who did not. This analysis aimed to identify any potential facilitators and barriers to screening participation in the study. The comparison revealed significant differences between both groups in the distribution of tobacco consumption (pack years) and disease involvement of neck nodes (regional disease) (Table 2).

### 3.3. Prevalence of Being at Risk of Oropharyngeal Dysphagia and Malnutrition

Of the 225 patients included in the study, 48 (21.3%) were identified as being at risk of OD, indicated by an EAT-10 score of ≥3. The overall median EAT-10 score was 0 (IQR: 0–2). The univariable analysis revealed significant differences in the overall median EAT-10 scores and in the number of patients scoring EAT-10 ≥ 3 for the characteristics of cancer stage grouping, primary tumor location, and BMI and SNAQ scores—both individually and as a composite measure for malnutrition risk (Table 3). More advanced cancer stages, tumors originating from the pharynx, abnormal BMI, SNAQ scores ≥ 2, and an overall higher malnutrition risk were associated with significantly higher median EAT-10 scores and a higher number of patients scoring ≥ 3 on the EAT-10.

**Figure 1:**
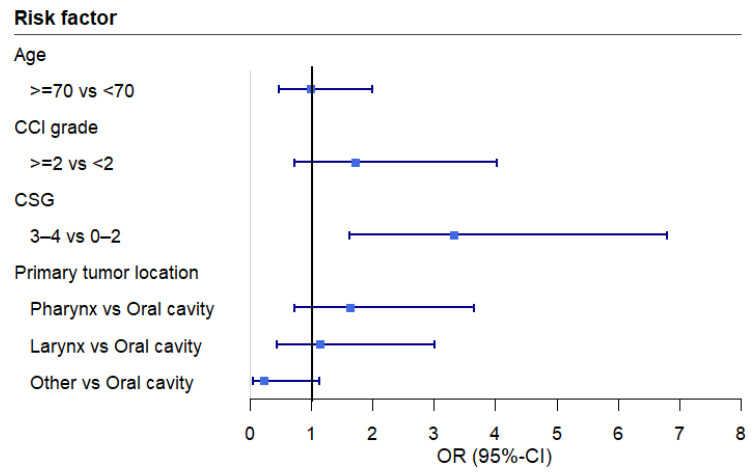
Forest plot of the odds ratios across the variables of the multivariable logistic regression analysis. Abbreviations: CCI: Charlson Comorbidity Index, CSG: cancer stage grouping, OR: odds ratio, CI: confidence interval.

**Figure 2:**
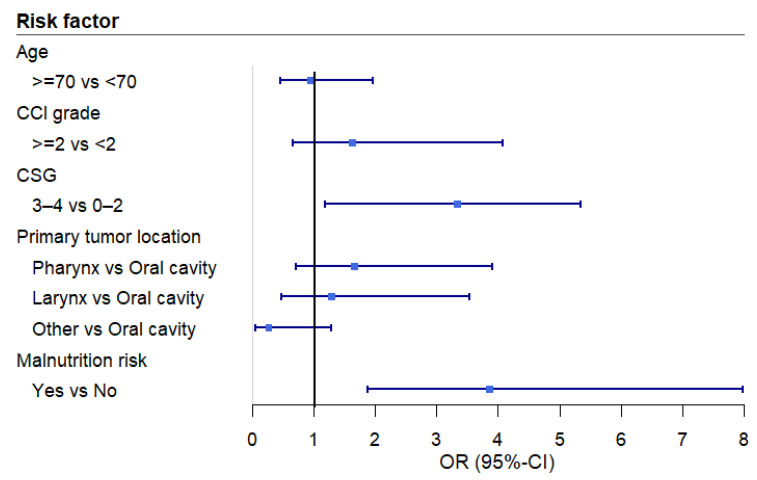
Forest plot of the odds ratios across the variables of the multivariable logistic regression analysis after correction for malnutrition. Abbreviations: CCI: Charlson Comorbidity Index, CSG: cancer stage grouping, OR: odds ratio, CI: confidence interval.

The multivariable analysis revealed a significant association between EAT-10 scores and cancer stage grouping (Table 4 and Figure 1). More advanced cancer stages were associated with an increased risk of OD (EAT-10 ≥ 3). This association remained significant even after applying a post hoc correction for malnutrition risk (Figure 2). To assess any potential loss of information due to the dichotomization, a sensitivity analysis was conducted. The sensitivity analysis, using linear regression, confirmed that advanced-stage cancer remained significantly associated with higher mean EAT-10 scores.

## 4. Discussion

In this prospective cohort study, we investigated the prevalence of being at risk of OD and its relationship with demographic and oncological characteristics among newly diagnosed HNC patients before the start of cancer treatment. The findings indicate that approximately one-fifth (21.3%) of all newly diagnosed HNC patients are at risk of OD (EAT-10 ≥ 3). This high prevalence is consistent with a smaller study, where about a quarter (26.2%) of the 128 HNC patients were at risk of OD (EAT-10 ≥ 3) before the start of cancer treatment [15].

The literature on the prevalence of OD at the time of HNC diagnosis, based on screening and clinical and/or instrumental assessments, is scarce [27,28]. Husmeela et al. (2021) reported an OD prevalence of 43.3% using the Modified Mann Assessment of Swallowing Ability scale [27]. However, their study population included both newly diagnosed HNC patients as well as those undergoing cancer treatment, making direct comparison with our study, in terms of study population, impossible. A meta-analysis by Porto de Toleda et al. (2019) investigated the prevalence of OD in newly diagnosed HNC patients using videofluoroscopic swallow study (VFSS) and fiberoptic endoscopic evaluation of swallowing (FEES) [28]. In this meta-analysis, 8.4% of HNC patients showed aspiration, 10.5% showed penetration, 16.0% had reduced laryngeal elevation, and 12.7% had pharyngeal residues before the start of cancer treatment. Due to the differences in patient populations and methodologies of the included studies, direct comparison between the findings of this meta-analysis and those of the present study is not possible.

In an ideal world with sufficient resources, all newly diagnosed HNC patients would undergo some form of comprehensive swallowing imaging in combination with patient-reported outcome measures on OD to determine the nature, severity, and burden of a baseline swallowing disorder [29,30]. However, given the large number of new patients, the required baseline diagnostic tests for cancer staging, and the SONCOS standard in the Netherlands—which mandates starting cancer treatment within 30 days of the first visit—swallowing imaging for all new HNC patients is not feasible. Therefore, we selected a screening method that identifies patients at risk of OD while ensuring health equity by screening all patients without increasing the diagnostic or financial burden.

The present study demonstrates a high prevalence of being at risk of OD, clearly emphasizing the importance of OD screening as an integrated part of the required baseline diagnostic trajectory for cancer staging. Screening allows for the timely identification of swallowing difficulties, enabling referral for subsequent diagnostic assessment at the interdisciplinary outpatient clinic for dysphagia, and swallowing (pre)habilitation to prevent complications such as aspiration pneumonia, malnutrition, and dehydration, ultimately improving oncological outcomes, including survival rates and overall health-related quality of life [6,31,32]. EAT-10 ≥ 3 before initiating upfront or postoperative chemoradiation for advanced-stage cancer has been shown to correlate with post-cancer treatment bolus aspiration and feeding tube dependency, suggesting that the EAT-10 not only highlights the prevalence of OD but also serves as a valuable indicator for OD-related complications following cancer treatment [14].

The descriptive statistics on the demographic and oncological characteristics of the newly diagnosed HNC patients revealed notable trends consistent with the existing literature, confirming the external validity of our study population. First, tumors originating in the oral cavity were among the most common primary tumor locations in the present study [1]. Secondly, the predominance of males in our study cohort reflects the well-documented gender disparity in the incidence of HNC. This disparity is largely attributed to differences in exposure to risk factors, particularly tobacco and alcohol consumption [33]. Additionally, the present study found a mean age of approximately 67 years at cancer diagnosis. Aging is a well-established risk factor for HNC, and older patients often present with more aggressive tumors and a higher likelihood of comorbidities [33].

To gain deeper insights into facilitators and barriers to screening participation in our study, a comparative analysis was conducted between patients who completed the EAT-10 questionnaire and those who did not. Patients who did not complete the EAT-10 questionnaire exhibited significantly higher levels of tobacco consumption and more advanced regional disease (N2–N3 classification). These findings suggest that patients with higher tobacco use and more advanced regional disease may be less likely to engage in OD screening. This may be due to factors like reduced health-seeking behavior commonly seen in patients with addictions, low awareness of OD and its consequences, socioeconomic challenges, or higher symptom burden related to advanced-stage cancer, all of which are barriers to adhering to cancer treatment as well [34,35]. Under Dutch privacy laws, patients are not required to provide a reason for opting out of screening, so the exact reasons for non-participation are not known for all patients. Additionally, nicotine suppresses appetite and increases metabolic rate, leading to a lower average weight in smokers, which may contribute to a more frail condition before the start of cancer treatment [35]. These findings suggest that non-participants require more attention and support from health professionals to encourage adherence to OD screening considering their higher risk of a frailer condition and advanced-stage cancer.

To optimize the screening framework for OD in HNC patients, this study investigated the associations between the prevalence of being at risk of OD before the start of cancer treatment and various baseline demographic and oncological characteristics. Of the four factors hypothesized to be associated with the risk of OD—older age, poor CCI grade, primary tumor location, and advanced-stage cancer—two were found to be statistically significant in the univariable analysis. Additionally, abnormal BMI, SNAQ scores ≥ 2, and overall malnutrition risk were significantly associated with higher median EAT-10 scores and with a higher proportion of patients scoring EAT-10 ≥ 3. These potential risk factors were subsequently included in the post hoc logistic regression analysis [6,36].

Advanced-stage cancer was found to be a significant risk factor for being at risk of OD. This finding is clinically understandable, as patients with more advanced-stage cancer may have a more affected upper aerodigestive tract, caused by the primary tumor and/or extensive regional disease invading important cranial nerves, muscles, and other structures necessary for swallowing [36]. Moreover, advanced-stage cancer at baseline may be associated with increased metabolic inflammation, cancer-induced catabolism, and skeletal muscle wasting, all of which can lead to OD [15,37]. This concept is reinforced by the occurrence of OD in newly diagnosed patients with other cancers outside of the head-and-neck region [38,39].

Finally, being at risk of malnutrition was also found to be a significant risk factor for being at risk of OD. This suggests that, in clinical practice, either an abnormal BMI (<20 kg/m^2^ if <70 years or <22 kg/m^2^ if ≥70 years old) or an elevated SNAQ score (≥2) should alert health professionals to the heightened risk of OD in HNC patients. From a clinical point of view, this finding makes sense, as it has been suggested that malnutrition may affect swallowing function due to sarcopenia and a malnourished swallowing muscle status, and OD and malnutrition are likely to interrelate [9,10]. To our knowledge, no research has yet examined the association between the risk of OD and malnutrition in baseline screenings, i.e., before the start of cancer treatment, of all newly diagnosed HNC patients, irrespective of cancer stage, primary tumor location or cancer treatment modality. However, there is established evidence of such an association in HNC patients post-cancer treatment [29,40], as well as in the general population [9,41,42,43]. Although the directionality of this association remains unclear—whether malnutrition primarily affects swallowing due to sarcopenia and a malnourished muscle status, or whether OD leads to malnutrition by reducing oral intake—the current finding underscores the importance of early nutritional screening, subsequent nutritional assessment, and nutritional intervention strategies in managing HNC patients [44].

The present prospective cohort study is limited by its sample size, which was insufficient to encompass all primary tumor sites, CCI grades, and cancer stages as individual categories of the variables in the multivariable logistic regression models. Consequently, not all clinically relevant associations may have been detected in this study.

The demographic and oncological characteristics of the EAT-10 participant sample aligned with existing literature, confirming the external validity of the study population and supporting the generalizability of the study’s outcomes. Nonetheless, selection bias is present due to the informed choice patients made regarding EAT-10 participation (deciding whether they were willing and able to participate in screening). This selection bias persisted despite oncology nurses providing all patients with uniform information. Through the present study, we became aware of this issue and are now making greater efforts to encourage screening participation from this vulnerable group of patients with higher tobacco use and more advanced regional disease.

The EAT-10 consists of items related to functional health status and health-related quality of life. The internal consistency, test–retest reliability, and criterion-based validity, coupled with its user-friendly nature, have rendered the EAT-10 widely adopted in clinical practice [14,16,45,46,47]. While existing literature points to some psychometric limitations of the EAT-10 [48,49], it strongly supports its validity as a self-report tool for identifying patients at risk of OD across various at-risk populations, with limitations common to all patient-reported tools [47,50]. A recent literature review on the EAT-10 and its clinical utility concluded that it serves as a viable tool for OD screening, supported by its psychometric properties, straightforward scoring system, and affordability [6]. Variations across study findings on psychometric properties of the EAT-10 may be due to differences in the external validity of patient cohorts, with selection of a gold standard being an imperfect diagnostic test for validation or differences in how patients scored the translated versions of the EAT-10 [49,50,51]. Finally, OD screening is just one of several clinically relevant screening domains for patients with HNC [15]. Even in resource-limited settings, the EAT-10 can be easily administered, preferably by health professionals with substantial experience in managing HNC.

Future research should explore the consequences of multidomain screening, particularly its impact on long-term cancer outcomes, including health-related quality of life and survival. Additionally, further studies on the acceptability and identification of facilitators/barriers to screening participation and to referral to specific allied health and/or medical disciplines are needed.

## 5. Conclusions

To conclude, in this study we unveil that approximately one-fifth of all newly diagnosed HNC patients are at risk of OD before the start of cancer treatment. Advanced-stage cancer and being at risk of malnutrition are risk factors for an increased risk of OD. These findings empower health professionals toward more effective screening and management of a patient’s risk profile before the start of cancer treatment. Continuous efforts are needed for making OD and malnutrition screening more acceptable, more accessible, and less time-consuming in this vulnerable group of patients. Future studies should investigate the effects of OD and malnutrition screening on cancer treatment outcomes and health-related quality of life.

## Figures and Tables

**Table 1:** Inclusion and exclusion criteria.

Inclusion Criteria	Exclusion Criteria
Diagnosis of HNC between December 2021 and May 2023	Illiteracy or blindness
≥18 years of age	Incomplete EAT-10 questionnaire
Informed consent	Diagnosis of incurable HNC
	Diagnosis other than HNC (e.g., sarcoma, thyroid gland carcinoma, skin cancer, hematological malignancies, etc.)
	Refusal of oncological diagnostics/staging
	Cancer treatment or follow-up at other hospital

Abbreviations: HNC: head-and-neck cancer, EAT-10: Eating Assessment Tool-10.

**Table 2:** Frequency distributions, measures of central tendency, and group comparisons of demographic and oncological characteristics for patients who completed the EAT-10 (included patients) versus those who did not (excluded patients).

Variable		Completed EAT-10	Not Completed EAT-10	*p*-Value
Age, N		N = 225	N = 41	
	Age < 70 years, N (%)	136 (60.4%)	26 (63.4%)	0.244 _d_
	Age ≥ 70 years, N (%)	89 (39.6%)	15 (36.6%)	0.774 ^a^
	Mean (SD)	66.8 (10.7)	67.3 (9.9)	
Sex, N		N = 225	N = 41	
	Male	144 (64.0%)	24 (58.5%)	0.598 ^c^
	Female	81 (36.0%)	17 (41.5%)	
Tobacco consumption, N		N = 225	N = 41	
	Never	37 (16.4%)	6 (14.6%)	0.956 ^d^
	Former	106 (47.1%)	20 (48.8%)	**0.039** ^b^
	Current	82 (36.4%)	15 (36.6%)	
	Number of pack years of smoking, median (IQR)	25.5 (5.3–43.0)	36.0 (21.5–55.0)	
Alcohol consumption, N		N = 224	N = 40	
	Never	59 (26.3%)	8 (20.0%)	0.276 ^d^
	Former	34 (15.2%)	10 (25.0%)	0.805 ^b^
	Current	131 (58.5%)	22 (55.0%)	
	Number of alcoholic drinks per day, median (IQR)	1.0 (0.3–3.0)	0.7 (0.1–3.0)	
Marital status, N		N = 220	N = 38	
	Single	34 (15.5%)	13 (34.2%)	0.068 ^d^
	Married	136 (61.8%)	20 (52.6%)	
	Divorced	6 (2.7%)	2 (5.3%)	
	Widower	23 (10.5%)	2 (5.3%)	
	Partner but not married	21 (9.6%)	1 (2.6%)	
Occupation, N		N = 165	N = 30	
	Employed	66 (40.0%)	13 (43.3%)	0.875 ^d^
	Unemployed	22 (13.3%)	5 (16.7%)	
	Retired	73 (44.2%)	11 (36.7%)	
	Voluntary work	4 (2.4%)	1 (3.3%)	
CCI grade, N		N = 224	N = 40	
	0 No comorbidity	108 (48.2%)	18 (45.0%)	0.301 ^d^
	1 Mild comorbidity	73 (32.6%)	10 (25.0%)	
	2 Moderate comorbidity	34 (15.2%)	8 (20.0%)	
	3 Severe comorbidity	9 (4.0%)	4 (10.0%)	
PS, N		N = 224	N = 40	
	0 Asymptomatic	152 (67.9%)	24 (60.0%)	0.114 ^d^
	1 Symptomatic, fully ambulatory	37 (16.5%)	7 (17.5%)	
	2 Symptomatic, in bed <50% of the day	27 (12.1%)	4 (10.0%)	
	3 Symptomatic, in bed >50% of the day	8 (3.6%)	5 (12.5%)	
	4 Completely disabled, bedridden	0 (0%)	0 (0%)	
SNAQ, N		N = 220	N = 4	
	<2, N (%)	181 (82.3%)	2 (50.0%)	0.154 ^c^
	≥2, N (%)	39 (17.7%)	2 (50.0%)	0.086 ^e^
	Median (IQR)	0 (0–1.0)	1.5 (0.3–3.5)	
BMI, N		N = 224	N = 39	
	Not at risk of malnutrition: ≥20 kg/m^2^ if age < 70 y or ≥22 kg/m^2^ if age ≥ 70 y, N (%)	192 (85.7%)	34 (87.2%)	0.901 _d_
	At risk of malnutrition: <20 kg/m^2^ if age < 70 y or <22 kg/m^2^ if age ≥ 70 y, N (%)	32 (14.3%)	5 (12.8%)	0.799 ^a^
	Mean (SD)	25.8 (5.0)	25.6 (4.7)	
Primary tumor location, N (%)		N = 225 (84.6%)	N = 41 (15.4%)	
	Oral cavity	77 (34.2%)	12 (9.32%)	0.834 ^d^
	(Para)nasal cavity	16 (7.1%)	1 (2.4%)	
	Pharynx	70 (31.1%)	15 (36.6%)	
	Larynx	45 (20.0%)	10 (24.4%)	
	Salivary glands	12 (5.3%)	2 (4.9%)	
	Neck lymph node metastasis of unknown primary origin	5 (2.2%)	1 (2.4%)	
T classification, N (%)		N = 225 (84.6%)	N = 41 (15.4%)	
	T0–1	76 (33.8%)	18 (43.9%)	0.367 ^d^
	T2	58 (25.8%)	6 (14.6%)	
	T3	31 (13.8%)	5 (12.2%)	
	T4	60 (26.7%)	12 (29.3%)	
N classification, N (%)		N = 225 (84.6%)	N = 41 (15.4%)	
	N0	145 (64.4%)	21 (51.2%)	**0.007** ^d^
	N1	36 (16.0%)	7 (17.0%)	
	N2	27 (12.0%)	10 (24.3%)	
	N3	17 (7.6%)	3 (7.3%)	
M classification, N (%)		N = 223 (84.5%)	N = 41 (15.5%)	
	M0	222 (99.6%)	41 (100%)	0.667 ^d^
	M1 (curable oligometastatic disease)	1 (0.4%)	0 (0.0%)	
CSG, N (%)		N = 224 (84.5%)	N = 41 (15.5%)	
	0–1	84 (37.5%)	17 (41.5%)	0.324 ^d^
	2	47 (21.0%)	4 (9.8%)	
	3	30 (13.4%)	5 (12.2%)	
	4	63 (28.1%)	15 (36.6%)	
HPV/p16, N (%)		N = 58 (25.7% of 225)	N = 11 (15.9% of 41)	
	Positive	24 (41.4%)	6 (54.5%)	0.514 ^c^
	Negative	34 (58.6%)	5 (45.5%)	
EBV, N (%)		N = 13 (22.4% of 225)	N = 1 (7.1% of 41)	
	Positive	8 (61.5%)	0 (0%)	0.429 ^c^
	Negative	5 (38.5%)	1 (100.0%)	

Abbreviations: SD: standard deviation, IQR: interquartile range, CCI: Charlson Comorbidity Index, PS: performance status, SNAQ: Short Nutritional Assessment Questionnaire, BMI: body mass index, T classification: tumor classification, N classification: node classification, M classification: metastasis classification, CSG: cancer stage grouping, HPV: human papillomavirus, EBV: Epstein–Barr virus. ^a^ Independent samples *t*-test; ^b^ Mann–Whitney U-test; ^c^ Fisher’s exact test; ^d^ Pearson chi-square test; ^e^ median test (Yates’s Continuity Corrected Asymptomatic Significance). Bold values denote statistical significance at the *p* < 0.05 level.

**Table 3:** Univariable analysis on median EAT-10 scores and number of patients scoring EAT-10 ≥ 3 for demographic and oncological characteristics.

Groups	EAT-10 Median (IQR)	*p*-Value	EAT-10 ≥ 3, N (%)	*p*-Value
Age < 70 years (N = 136)	0 (0–2)	0.892 ^a^	29 (21.3)	0.996 ^b^
Age ≥ 70 years (N = 89)	0 (0–2)		19 (21.3)	
Male (N = 144)	0 (0–2)	0.312 ^a^	29 (20.1)	0.560 ^b^
Female (N = 81)	0 (0–2)		19 (23.5)	
CCI grade < 2 (N = 181)	0 (0–2)	0.982 ^a^	37 (20.3)	0.450 ^b^
CCI grade ≥ 2 (N = 43)	0 (0–4)		11 (25.6)	
PS 0–1 (N = 189)	0 (0–2)	0.247 ^a^	38 (20.1)	0.262 ^b^
PS 2–3 (N = 35)	0 (0–5)		10 (28.6)	
CSG 0–2 (N = 131)	0 (0–1)	**0.030** ^a^	17 (13.0)	**<0.001** ^b^
CSG 3–4 (N = 93)	0 (0–6)		31 (33.3)	
Oral cavity (N = 77)	0 (0–1)	**0.020** ^a^	14 (18.2)	**0.014** ^b^
Pharynx (N = 70)	0.5 (0–6.5)		23 (32.9)	
Larynx (N = 45)	0 (0–2)		9 (20.0)	
Other (N = 33)	0 (0–0)		2 (6.1)	
Normal BMI ^c^ (N = 193)	0 (0–1.5)	**0.004** ^a^	37 (19.2)	0.052 ^b^
Abnormal BMI ^c^ (N = 32)	1.5 (0–12)		11 (34.4)	
SNAQ < 2 (N = 181)	0 (0–1)	**<0.001** ^a^	26 (14.4)	**<0.001** ^b^
SNAQ ≥ 2 (N = 39)	3 (0–19)		20 (51.3)	
No malnutrition risk ^d^ (N = 161)	0 (0–1)	**<0.001** ^a^	22 (13.7)	**<0.001** ^b^
Malnutrition risk ^d^ (N = 61)	2 (0–11)		25 (41.0)	

Abbreviations: IQR: interquartile range, CCI: Charlson Comorbidity Index, PS: performance status, CSG: cancer stage grouping, BMI: body mass index, SNAQ: Short Nutritional Assessment Questionnaire. ^a^ Median test (Yates’s Continuity Corrected Asymptomatic Significance); ^b^ Pearson chi-square test; ^c^ abnormal BMI: <20 kg/m^2^ if <70 years or <22 kg/m^2^ if ≥70 years old; ^d^ malnutrition risk was defined by SNAQ and BMI: SNAQ ≥ 2 or abnormal BMI. No malnutrition risk: SNAQ < 2 and normal BMI. Bold values denote statistical significance at the *p* < 0.05 level.

**Table 4:** Multivariable logistic regression analysis of patients scoring EAT-10 ≥ 3 with the variables of age, CCI grade, cancer stage grouping, and primary tumor location as risk factors, including post hoc correction for malnutrition risk.

Variable	OR (95% CI)	*p*-Value	Post Hoc OR (95% CI)	Post Hoc *p*-Value
Age				
<70 years	Reference	0.945	Reference	0.861
≥70 years	0.98 (0.48, 1.99)		0.94 (0.45, 1.96)	
CCI grade				
<2	Reference	0.224	Reference	0.292
≥2	1.71 (0.72–4.03)		1.63 (0.66–4.07)	
CSG				
0–2	Reference	<0.001	Reference	0.016
3–4	3.33 (1.63–6.80)		3.33 (1.19–5.35)	
Primary tumor location		0.090		0.121
Oral cavity	Reference		Reference	
Pharynx	1.63 (0.72–3.66)	0.238	1.66 (0.71–3.90)	0.243
Larynx	1.14 (0.43–3.01)	0.788	1.29 (0.47–3.53)	0.615
Other	0.23 (0.05–1.14)	0.072	0.26 (0.05–1.28)	0.097
Malnutrition risk ^a^				
No			Reference	<0.001
Yes			3.86 (1.87–7.99)	

Abbreviations: OR: odds ratio, CI: confidence interval, CCI: Charlson Comorbidity Index, CSG: cancer stage grouping. ^a^ Abnormal BMI: <20 kg/m^2^ if <70 years or <22 kg/m^2^ if ≥70 years old. Malnutrition risk was defined by SNAQ and BMI: SNAQ ≥ 2 or abnormal BMI. No malnutrition risk: SNAQ < 2 and normal BMI.

## Data Availability

The data that support the findings of this study are available upon request from the corresponding author. The data are not publicly available due to privacy restrictions.
